# Ionic Conductive Hydrogels with Choline Salt for Potential Use in Electrochemical Capacitors

**DOI:** 10.3390/polym17223030

**Published:** 2025-11-14

**Authors:** Jan Malczak, Wiktoria Żyła, Piotr Gajewski, Katarzyna Szcześniak, Łukasz Popenda, Agnieszka Marcinkowska

**Affiliations:** 1 Institute of Chemical Technology and Engineering, Poznan University of Technology, Berdychowo 4, 60-965 Poznan, Poland; jan.malczak@student.put.poznan.pl (J.M.);; 2 NanoBioMedical Centre, Adam Mickiewicz University, Wszechnicy Piastowskiej 3, 61-614 Poznan, Poland

**Keywords:** choline salt, choline methacrylate, hydrogel polymer electrolyte, photopolymerization, capacitor, puncture resistance

## Abstract

Choline salts represent sustainable and safe electrolyte systems. In this study, an aqueous 1 M choline nitrate solution was employed to prepare hydrogel polymer electrolytes (HPE) via in situ photopolymerization. To enhance compatibility between the electrolyte and polymer matrix, choline methacrylate was synthesized and used as a functional monomer alongside HEMA and PEGDA. The photocurable formulation contained 70 wt.% electrolyte and 30 wt.% monomer mixture. Subsequent electrolyte uptake increased the electrolyte fraction in the HPE to 87 wt.%. The use of choline methacrylate enabled the formation of transparent HPE with favorable mechanical performance, showing puncture resistance of 0.33 N and 0.28 N at elongations of 7.9 mm and 4.4 mm for samples with 70 and 87 wt.% electrolyte, respectively. High ionic conductivity was achieved, reaching ~18 mS/cm and ~34 mS/cm for HPE with 70 and 87 wt.% electrolyte. Finally, a capacitor assembled with HPE containing 87 wt.% electrolyte demonstrated good operational parameters, confirming the applicability of this system in energy storage devices. This work highlights the potential of choline-based electrolytes and polymerizable choline derivatives as functional components for the design of efficient, safe, and environmentally friendly gel polymer electrolytes.

## 1. Introduction

In recent years, global demand for energy derived from renewable sources has grown rapidly. Renewable energy generation, particularly from solar photovoltaics and wind power, is inherently intermittent and variable: photovoltaic systems operate only during daylight hours, and wind output fluctuates depending on weather conditions. Since such sources do not provide continuous or easily dispatchable power, reliable energy storage technologies are essential to balance energy supply and demand. A wide range of storage systems have been explored, differing in both scale and operating mechanisms. Among them, electrochemical energy storage—including batteries and supercapacitors—is especially attractive due to its high efficiency, fast response, modular design, and scalability across diverse applications [1,2].

Electrochemical capacitors (ECs), often referred to as supercapacitors or ultracapacitors, are particularly effective in applications requiring high power density; that is, the ability to deliver or absorb large currents within a short space of time. They bridge the gap between conventional dielectric capacitors, which provide extremely high power but low energy density, and batteries, which offer the reverse—high energy but limited power output [2,3]. In ECs, the electrolyte, along with electrode materials and separator, plays a crucial role by enabling ion transport between electrodes during charge and discharge. As a result, the capacitance, power density, and cycling stability of ECs are strongly dependent on the electrolyte composition and its properties [4]. Traditionally, supercapacitors have employed liquid electrolytes, either aqueous (acids, bases, or salt solutions) or non-aqueous (organic solvents with dissolved salts), depending on the desired voltage window and conductivity. Although such electrolytes ensure excellent ionic mobility and good electrode wetting, they also come with several drawbacks [3,5,6]. Leakage of the liquid phase, for instance, can lead to the release of corrosive, toxic, or flammable substances, posing significant safety and environmental risks, especially for portable and large-scale applications [7]. Strongly acidic or basic aqueous systems may corrode current collectors and cell components, limiting long-term durability [8]. Organic electrolytes, while offering wider voltage windows, often rely on volatile, flammable solvents that may evaporate over time, shortening device lifetime. Even ionic liquids, though non-volatile, often suffer from high viscosity and reduced ionic mobility at low temperatures [6]. These limitations have driven the search for safer electrolytes that combine good electrochemical performance with improved stability and reduced risk of leakage or flammability [9]. One promising direction involves gel polymer electrolytes (GPEs), which integrate the ionic conductivity of liquids with the mechanical stability and formability of polymers. GPEs consist of a polymer matrix swollen with a liquid electrolyte (aqueous, organic, ionic liquid, or deep eutectic solvent) [10,11]. Their semi-solid nature prevents free-liquid leakage while maintaining high ionic transport, and their flexibility makes them suitable for emerging applications such as flexible or wearable devices [10,12,13,14]. Depending on the composition, GPEs can be tailored with functional ions or additives to enhance performance—one example being the use of choline-based species.

Choline (2-hydroxyethyltrimethylammonium) is a quaternary ammonium cation that occurs naturally in biological systems and has broad industrial use. It is commonly available as salts, such as choline chloride, or as derivatives. Choline-based salts are of interest for electrolyte development because they are inexpensive, biodegradable, highly water-soluble, and exhibit low toxicity compared with many conventional ionic liquid cations. They are frequently used as components of deep eutectic solvents (DESs) and ionic liquids [15,16]. Choline-based electrolytes—including choline nitrate [17,18], choline chloride [19], various DESs [2], and choline-containing ionic liquids [20]—have been explored for energy storage applications, showing low freezing points (down to −40 °C) and favorable environmental profiles [17,21]. For example, Supiyeva et al. [17] reported a carbon–carbon supercapacitor employing a choline-based electrolyte (5 mol/kg with 10 vol.% methanol) which achieved a specific capacitance of 103 F/g at room temperature and retained 78 F/g at −40°C, demonstrating the excellent low-temperature performance achievable with choline salts.

A logical next step in harnessing the advantages of choline-based electrolytes is to develop polymeric systems that maintain high ionic conductivity while improving mechanical strength and stability. Choline methacrylate (ChMAA) is a methacrylate monomer containing a choline cationic group, which can be polymerized via conventional radical or photopolymerization methods to form poly (ionic liquid)-type networks or copolymers. Such materials incorporate ionic sites directly into the polymer backbone, allowing for control over counter-ion composition and thereby tuning conductivity and electrochemical behavior. Polymers containing choline and methacrylate moieties have already been studied in biomedical and drug-delivery applications, where anion exchange enables the incorporation of active species [22,23]. Their demonstrated versatility and biocompatibility make ChMAA-based systems promising candidates for gel polymer electrolytes in supercapacitors. However, despite this potential, reports on the electrochemical performance of ChMAA-based hydrogels in full EC devices remain scarce, indicating a clear opportunity for further investigation.

In this work, gel polymer electrolytes were synthesized via in situ photopolymerization using methacrylate-based monomers with the addition of choline methacrylate. The introduction of this functional monomer aimed to improve the compatibility between the polymer matrix and an aqueous choline salt electrolyte. The use of an aqueous choline nitrate solution as the ionic conductor provides an environmentally friendly and low-toxicity alternative to conventional electrolytes. The resulting system combines good ionic conductivity with mechanical integrity, offering potential for safe and sustainable applications in electrochemical energy storage devices.

## 2. Materials and Methods

### 2.1. Materials

Monomer synthesis: methacrylic acid (MAA, 99% Sigma Aldrich, St. Louis, MO, USA), choline chloride (ChCl, ≥98% Sigma Aldrich, St. Louis, MO, USA), sodium hydroxide (98.8%, Chempur, Piekary Śląskie, Poland), ethanol (EtOH, Sigma Aldrich, 96%).

Hydrogel polymer electrolyte synthesis: poly (ethylene glycol) diacrylate (PEGDA, Mw = 545 g·mol^−1^, Sigma Aldrich, 99%, St. Louis, MO, USA), 2-hydroxyethyl methacrylate (HEMA, Sigma Aldrich, 97%, St. Louis, MO, USA), 1-hydroxycyclohexylphenylketone (Irgacure 184, 99.9%BASF, Ludwigshafen, Germany), choline nitrate (ChNO_3_, synthesized in-house).

### 2.2. Methods

#### 2.2.1. Choline Methacrylate (ChMAA) Synthesis

Sodium hydroxide (0.1 mol, 4.000 g) was dissolved in ethanol (100 mL), and methacrylic acid (0.1 mol, 8.609 g) was mixed with ethanol (100 mL). Both solutions were poured into a conical flask in an equimolar ratio of NaOH to MAA and stirred on a magnetic stirrer (1000 rpm) at room temperature to obtain sodium methacrylate (NaMAA). The reaction proceeded according to Equation (1).
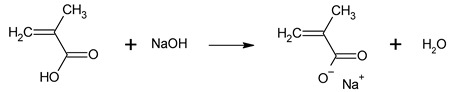
(1)

Choline chloride (0.1 mol, 13.962 g) dissolved in ethanol (100 mL) was then added to the reaction vessel with sodium methacrylate in a 1:1 molar ratio, assuming that the synthesis of sodium methacrylate was 100% efficient. The reaction mixture was left on a magnetic stirrer at room temperature for approximately 8 h. The ion exchange reaction proceeded according to Equation (2).
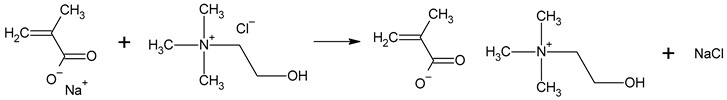
(2)

In the next step, the liquid phase containing the ChMAA monomer in ethanol was separated from the NaCl precipitate by filtration through filter paper. The solvent was evaporated from the liquid phase using a vacuum evaporator, and the resulting ChMAA monomer was dissolved in a small amount of ethanol. The solution was filtered through a syringe filter to remove any impurities. The ethanol was again distilled using a vacuum evaporator, and the monomer was placed in a desiccator containing P_2_O_5_ drying agent to remove water. The synthesized ChMAA monomer was stored in a dark container under refrigerated conditions at 4 °C.

#### 2.2.2. Preparation of Photocurable Compositions

Appropriate amounts of monomers (ChMAA, PEGDA, HEMA) were weighed into a glass vial to constitute 30 wt.% of the light-cured composition. Each composition contained 10 wt.% of the synthesized ChMAA monomer. The remaining 70 wt.% of the photocurable composition was the solvent, i.e., a 1M aqueous solution of choline nitrate. The photoinitiator was added to the mixture at a rate of 0.2 wt.% of the total composition weight. The system was mixed using an orbital shaker until all components dissolved and a homogeneous mixture was obtained. The composition of the individual mixtures is presented in Table 1.

#### 2.2.3. Hydrogel Polymer Electrolyte Synthesis

**Scheme 1:**
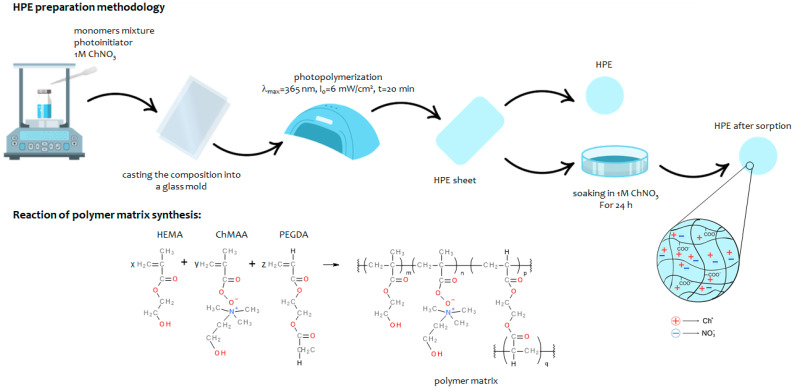
Schematic illustration of the methodology applied for gel preparation, structure of polymer matrix and schematic illustration of hydrogel structure.

The prepared photocurable composition was polymerized at room temperature in a UV lamp (Evershine, SP-16, 36 W) at an intensity of 6 mW·cm^−2^, in the form of sheets in a glass mold (dimensions 10 cm × 10 cm × 300 μm) covered with Teflon foil to prevent HPE adhesion. The exposure time was 20 min. From the finished HPE sheet, 12 mm diameter samples were cut to test the ionic conductivity and 13 mm diameter samples were cut to test the puncture resistance. Additionally, HPE sheet was placed in an aqueous solution of 1 M ChNO_3_ for approximately 24 h, and then samples with 12 and 13 mm diameters were cut again for further testing. The method of HPE preparation is shown in Scheme 1.

#### 2.2.4. FTIR-ATR Spectroscopy

The IR spectrum of the ChMAA monomer was obtained using a Nikolet 5700 Fourier Transform Infrared Spectrophotometer (Thermo Elecetron, Scientific Instruments Corporation, Madison, WI, USA) equipped with a diamond ATR attachment. The study was performed at room temperature in the wavenumber range of 4000–400 cm^−1^ with a resolution of 4 cm^−1^.

#### 2.2.5. NMR

The NMR measurements were performed by means of Agilent DD2 800 spectrometer (Agilent Technologies, Santa Clara, CA, USA) equipped with a 5 mm 1H{13C/15N} probe head. The 1D (^1^H, ^13^C) and 2D (^1^H-^1^H COSY, ^1^H-^13^C HSQC, ^1^H-^13^C HMBC) experiments were carried out at 25 °C in DMSO-*d*_6_/D_2_O (5:1, *v*/*v*). Data were processed and analyzed with MestReNova 15 software. Chemical shift values were given in ppm and referenced to TMS (δ_H_ = 0.00 ppm, δ_C_ = 0.00 ppm), used as an internal standard.

#### 2.2.6. Electrolyte Sorption

The HPE sheet was weighed after photopolymerization and placed in a 1M ChNO_3_ aqueous solution. After 24 h, the sample was removed from the electrolyte, dried to remove excess electrolyte using absorbent paper, and weighed again. The total percentage of electrolyte (*X_El_*) in the HPE mass was calculated from Equation (3).

(3) $$X_{El}=\frac{m_{2}-0.3\cdot m_{1}}{m_{2}}\cdot 100\%$$

where *X_El_*—electrolyte content in HPE [%], *m*_1_—HPE mass before electrolyte absorption [g], *m*_2_—HPE mass after electrolyte absorption [g], 0.3—matrix content in hydrogel.

#### 2.2.7. Thermal Characteristics

The crystallization and melting temperatures of the electrolyte and the hydrogels synthesized in this work were determined using differential scanning calorimetry on a DSC 1 apparatus (Mettler Toledo, Greifensee, Switzerland). A 5–10 mg sample of electrolyte or hydrogel was placed in a DSC crucible, which was then pressed with a lid. A hole was made in the lid to allow free evaporation of volatile compounds. The sample was then placed in the apparatus chamber and heated/cooled at a rate of 10 °C·min^−1^. The inert gas (argon) flowed through the chamber at a rate of 20 mL·min^−1^, and the shielding gas (nitrogen) flowed at a rate of 100 mL·min^−1^. The measurements were performed in the temperature range from 25 °C to −50 °C.

#### 2.2.8. Puncture Resistance

The puncture resistance of HPE was measured using a Brookfield CT3 Texture Analyzer (Ametek Brookfield, Middleboro, MA, USA). For testing HPE samples with a diameter of 13 mm were used. Their thickness was measured and mounted in a measuring fixture with a 10 mm diameter opening. A 5 mm diameter spherical measuring probe moved at a speed of 0.3 mm·s^−1^. The minimum force was 0.02 N. During the measurement, the force exerted on the sample and the displacement of the measuring probe until the test sample punctured were recorded. The obtained results were used to determine the puncture elongation and the force required to puncture the sample. The results were then normalized to a constant HPE thickness of 300 μm.

#### 2.2.9. Ionic Conductivity

The ionic conductivity of the prepared hydrogel polymer electrolytes was studied by electrochemical impedance spectroscopy in the frequency range from 1 kHz to 1 MHz using a Biologic SP-300 potentiostat (Biologic, Seyssinet-Pariset, France). The studies were performed in a Swagelok^®^ (Swagelok, Solon, OH, USA) two-electrode electrochemical cell between two 316L stainless steel collectors at room temperature. For each sample, the study was conducted both before and after electrolyte absorption. The ionic conductivity (HPE) was calculated using Equation (4).

(4) $$\sigma =\frac{l}{A}\cdot \sigma_{s}$$

where *σ*—ionic conductivity [S·cm^−1^], *l*—sample thickness [cm], *A*—sample surface [cm^2^], *σ_s_*—conductivity of tested HPE [S].

The ionic conductivity of the liquid electrolyte—1 M ChNO_3_—was measured at room temperature using an electrochemical cell designed for liquid samples and calculated using Equation (5).

(5) $$\sigma =k\cdot \sigma_{s}$$

where *σ*—ionic conductivity [S·cm^−1^], *k*—cell constant equal to 1.54 [cm^−1^], *σ_s_* –conductivity of tested electrolyte [S].

Additionally, a temperature-dependent ionic conductivity study was conducted. Measurements were performed in a BINDER MK56 climatic chamber at temperatures ranging from 25 °C to −40 °C. Measurements were taken every 5 °C. HPE samples were placed in a Swagelok^®^ measuring system between two 316L stainless steel collectors. HPE ionic conductivity was calculated using Equation (4).

#### 2.2.10. Preparation of Electrodes and Electrochemical Capacitor

Carbon-based electrodes were obtained by mixing 85 wt.% activated carbon (Maxsorb MSP-20X, Kansai Coke and Chemicals Co., Hyogo, Japan) with 5 wt.% carbon black (C65, Imerys) and 10 wt.% thermoplastic polyurethane (TPU) in *N*,*N*-dimethylformamide (DMF) as the solvent. The components were thoroughly mixed in a vial until a homogeneous suspension was obtained. The resulting slurry was cast onto a glass substrate using a film applicator, followed by solvent evaporation under ambient conditions. The electrode films were subsequently dried in an oven at 70 °C for 12 h to ensure complete removal of residual solvent. After drying, the films were carefully detached from the substrate, and circular electrodes with a diameter of 12 mm were punched out. The prepared electrodes were then attached to 316L stainless-steel current collectors using Electrodag PF-407C conductive adhesive (Henkel, Dusseldorf, Germany).

Symmetric two-electrode supercapacitor cells were assembled in a Swagelok^®^ configuration, employing 316L stainless steel as the current collectors. Prior to assembly, the electrodes were impregnated with the electrolyte, and a synthesized gel was used as the separator. The average mass of activated carbon per electrode was approximately 9 mg, and this value was used for current density and specific capacitance calculations.

Electrochemical characterization was carried out using a two-electrode Swagelok^®^-type capacitor cell connected to an SP-300 potentiostat/galvanostat (Biologic, Seyssinet-Pariset, France). The performance of the devices was evaluated by cyclic voltammetry (CV) at scan rates ranging from 5 to 100 mV·s^−1^ within a potential window of 0.8–1.5 V, and by galvanostatic charge–discharge (GCPL) measurements at current densities between 0.5 and 4 A·g^−1^, calculated based on the average mass of activated carbon in a single electrode. Electrochemical impedance spectroscopy (EIS) was performed at open-circuit potential over a frequency range from 1 MHz to 1 mHz with a sinusoidal perturbation amplitude of 7 mV. The specific energy of the EC was measured within a voltage range of 1.50–0.75 V and in the specific power range of 150–8500 W·kg^−1^. Specific energy and specific power were calculated per total mass of both electrodes. The self-discharge behavior was evaluated by charging the capacitor to 1.50 V and monitoring the open-circuit voltage for 4 h. The long-term stability of the electrochemical capacitor was evaluated by accelerated aging involving 30 repeated cycles of galvanostatic charge/discharge and 2 h potentiostatic “floating” periods at 1.5 V, corresponding to a total floating time of 60 h. Each floating step was preceded by five galvanostatic cycles at 1 A·g^−1^. The capacitance and efficiency were determined from the fifth charge/discharge. The normalized value of capacitance C/C_0_ was used to assess the state of health (SOH) of the capacitors.

## 3. Results and Discussion

In this study, an aqueous electrolyte based on a choline salt (choline nitrate) was selected to obtain a material with a lowered freezing point while maintaining environmentally benign properties. Such an approach may help prevent environmental contamination in the event of electrolyte leakage from an energy storage device and reduce potential adverse health effects for the user. To the best of our knowledge, aqueous choline salt solutions have not been explored as electrolytes in gel polymer electrolyte systems. In contrast, most studies report the use of choline-based deep eutectic solvents as electrolytes [24,25,26,27,28], leading to the formation of organogels, also known as eutectogels. In our work, the use of an aqueous choline electrolyte results in the formation of a hydrogel-type polymer electrolyte. The choline nitrate water solution we used in this study acts both as a solvent and as an ion carrier. The experimental plan involved the in situ synthesis of a hydrogel polymer electrolyte via photopolymerization. However, significant challenges were encountered, as the electrolyte showed poor miscibility with the methacrylate monomers typically used for hydrogel preparation. Various monomers, including HEMA, HEMA phosphate, 2-hydroxylpropyl methacrylate, PEGDA, trimethylolpropane triacrylate (TMPTA), 1,6-hexanediol diacrylate (HDDA), as well as resins such as Ebecryl 4858 or Exothane 8, were investigated. Both difunctional and multifunctional monomers were selected in order to obtain hydrogels with a controlled and appropriate network density. Difunctional monomers, such as HEMA, tend to polymerize to form linear polymer chains, which provide flexibility to the system. In contrast, multifunctional monomers, such as PEGDA, HDDA, TMPTA contain at least two reactive groups within a single molecule, allowing the formation of a three-dimensional crosslinked polymer network. The careful selection of the chemical structure and functionality of the monomers enables the formation of a well-developed polymer matrix capable of effectively entrapping the solvent within the hydrogel structure. Such a crosslinked network ensures the mechanical stability of the gel and prevents liquid electrolyte leakage during use. Among these, HEMA and PEGDA yielded the most promising results; nevertheless, their miscibility with the electrolyte remained limited, and the obtained polymer hydrogels exhibited pronounced syneresis accompanied by electrolyte leakage. While polymethacrylates are generally well compatible with water, the introduction of choline nitrate as an ion carrier altered the interactions within the aqueous medium, leading to phase separation between the gel components and, consequently, destabilization of the hydrogel network manifested by electrolyte release. Consequently, it was decided to design and synthesize a choline-based monomer, which is expected to enhance the miscibility of the electrolyte both with the monomer mixture and within the resulting polymer hydrogel.

### 3.1. Monomer Synthesis

To improve compatibility between the polymer matrix and the electrolyte, choline methacrylate (ChMAA) was synthesized and used as one of the monomers in the hydrogel polymer synthesis. The synthesis of choline methacrylate was performed according to the research methodology presented in Section 2.1, and its structure was confirmed by FTIR and NMR methods.

**Figure 1:**
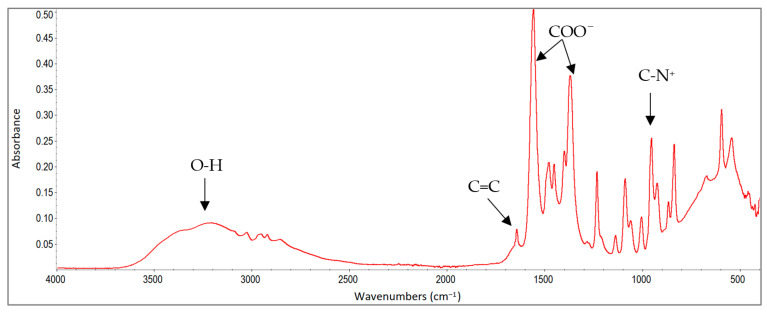
FTIR-ATR spectrum of choline methacrylate (ChMAA) (diamond crystal, room temperature, resolution 4 cm^−1^).

The infrared spectroscopy spectrum shown in Figure 1 exhibits absorption bands, confirming the presence of characteristic functional groups in the obtained ChMAA compound. A broad absorption band in the wavenumber range of 3600–3000 cm^−1^ corresponds to the stretching vibrations of the hydroxyl (-OH) group in the choline cation. The band with the maximum absorption at wavenumber 1643 cm^−1^ corresponds to the stretching vibrations of the unsaturated methacrylic group C=C bond. Bands indicating the carboxylate group are visible in the spectrum at wavenumbers of 1557 cm^−1^ (asymmetric vibrations) and 1366 cm^−1^ (symmetric vibrations), confirming the ionic nature of this functional group. Additionally, a stretching vibration band of the C-N_+_ quaternary ammonium group of choline is visible at wavenumber 953 cm^−1^, as well as bending vibrations of the alkanes C-H (-CH_2_) in the ranges of 3000–2800 cm^−1^ and 1481–1453 cm^−1^.

**Figure 2:**
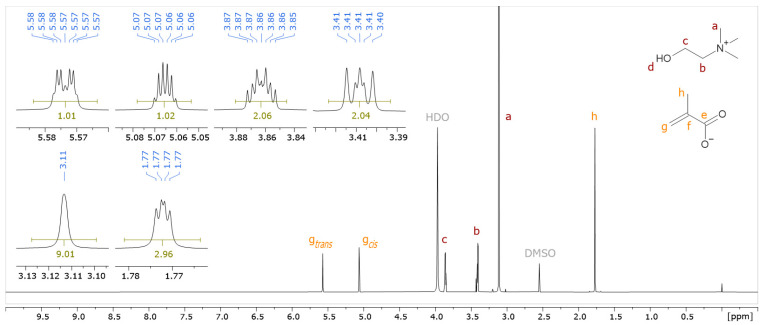
^1^H NMR spectrum of choline methacrylate; the structure of the monomer with signal assignments and the measured spectrum (DMSO-*d*_6_/D_2_O, 5:1; 25 °C, internal standard TMS).

**Figure 3:**
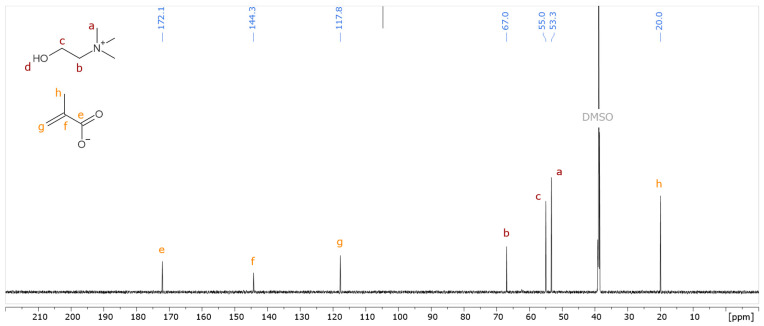
^13^C NMR spectrum of choline methacrylate; the structure of the monomer with signal assignments and the measured spectrum (DMSO-*d*_6_/D_2_O, 5:1; 25 °C, internal standard TMS).

The synthesized monomer was determined on the basis of ^1^H NMR (Figure 2) and ^13^C NMR (Figure 3) spectra. The results of the NMR measurements are as follows:

^1^H NMR (800 MHz, DMSO-*d*_6_/D_2_O 5:1 (*v*/*v*)): δ 5.57 (dq, *J* = 3.1, 0.9 Hz, 1H, C=CH_2_), 5.07 (dq, *J* = 3.1, 1.6 Hz, 1H, C=CH_2_), 3.88–3.85 (m, 2H, CH_2_OH), 3.42–3.39 (m, 2H, NCH_2_), 3.11 (s, 9H, N(CH_3_)_3_), 1.77 (dd, *J* = 1.6, 0.9 Hz, 3H, CH_3_), ^13^C NMR (201 MHz, DMSO-*d*_6_/D_2_O 5:1 (*v*/*v*)): δ 172.1, 144.3, 117.8, 67.0, 55.0, 53.3, 20.0.

### 3.2. Hydrogel Polymer Electrolytes Synthesis

The choice of polymer matrix proved decisive for the performance of the gel systems. Since polymerization was required to occur directly in the electrolyte, only components soluble in water solution of ChNO_3_ could be considered, which ruled out many monomers and resins typically compatible with organic solvents, as was mentioned above. Crosslinking monomer PEGDA was ultimately selected, as it satisfied this solubility criterion and enabled the formation of stable gels. Mechanical performance was also a key factor: the materials had to retain structural integrity while providing flexibility, resistance to bending, and low brittleness. Limited phase separation was found to be beneficial, as it could enhance ionic conductivity by weakening intermolecular interactions, although both complete separation and its absence were still acceptable for further testing.

In addition to the monomers shown in Table 1, a variety of different monomers and oligomers were initially tested to assess their suitability for HPE synthesis. Most of prepared formulations proved unsuitable due to immiscibility with the electrolyte or poor mechanical stability after polymerization. Nevertheless, this preliminary evaluation was essential, as it led to the identification of gel systems with the desired balance of solubility, stability, and mechanical properties, which were subsequently selected for further investigation.

**Figure 4:**
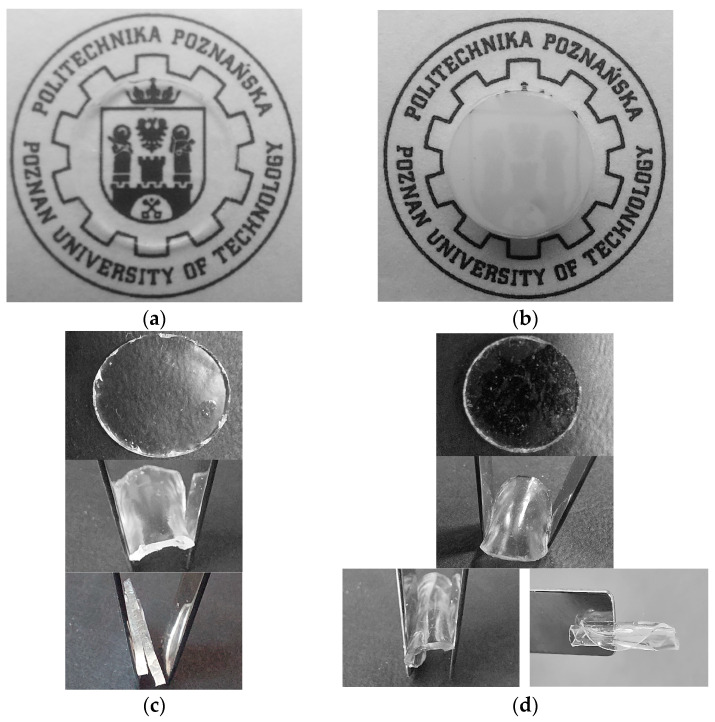
Visual comparison of obtained hydrogel polymer electrolytes: (**a**) transparent HPE_4, showing good component compatibility, (**b**) opalescent gel synthesized without the addition of ChMAA monomer, indicating partial phase separation, (**c**) brittle HPE_2 gel with limited flexibility; and (**d**) elastic HPE_4 gel demonstrating high mechanical integrity.

Both the prepared photocurable compositions and the obtained gel samples were subjected to a preliminary visual and tactile evaluation, including assessment of transparency, homogeneity, elasticity, and brittleness, to determine their overall quality and integrity. According to the literature [29] fully transparent hydrogels typically exhibit higher mechanical strength than opalescent ones, as the latter possesses a multiphase structure with visible structural imperfections. The presence of interfaces between phases acts as stress concentrators, facilitating crack initiation and propagation under mechanical load, which consequently reduces the overall strength of the material. It is also worth mentioning that when a good solvent for polymer is used, the interactions between the solvent molecules are less favorable than the interactions between the solvent molecules and the polymer matrix. Therefore, the stronger the interactions between the gel components, the greater the probability of obtaining a transparent gel with good mechanical properties. The compositions listed in Table 1 were fully miscible prior to polymerization, forming homogeneous solutions. After polymerization, the resulting HPE_1–HPE_6 polymer gel electrolytes were transparent, as illustrated by the HPE_4 sample in Figure 4a. For comparison, Figure 4b presents a gel polymer electrolyte prepared without the ChMAA monomer, where incomplete compatibility between the components leads to the formation of structural domains that scatter light, resulting in an opalescent appearance. This effect originates from phase separation, which creates distinct regions within the material that differ in density and composition. The numerous interfaces between these regions cause light scattering, thereby hindering clear transmission through the sample while allowing partial passage of light—a characteristic feature of translucent systems. Following polymerization, samples HPE_1 and HPE_2 exhibited no visible phase separation; however, they were characterized by brittleness and limited elasticity (Figure 4c). Reducing the concentration of the crosslinking monomer PEGDA to 1.5 wt.% in favor of increasing the HEMA content (sample HPE_3) led to a marked improvement in elasticity, although the material still retained a brittle character. A further decrease in PEGDA concentration to 1.2 wt.% (sample HPE_4) resulted in a polymer gel system with the desired characteristics, namely transparency, flexibility, and the absence of brittleness (Figure 4d). In contrast, additional reduction in PEGDA content in samples HPE_5 and HPE_6 produced materials with insufficient crosslinking density, which prevented the gels from maintaining structural stability. Therefore, a hydrogel polymer electrolyte HPE_4, presented on Figure 4, containing 10 wt.% ChMAA, 1.2 wt.% PEGDA, 18.8 wt.% HEMA and 70 wt.% 1M ChNO_3_ aqueous solution was selected for further studies.

### 3.3. Sorption of Electrolyte

Electrolyte absorption testing was conducted for the HPE_4 sample to determine the maximum electrolyte uptake capacity of the prepared hydrogel polymer electrolyte. The electrolyte content in HPE_4 increased from 70 wt.% to 87 wt.%, which indicates the presence of strong interactions between the polymer matrix and the aqueous electrolyte. This is also evidenced by the lack of phase separation in the system, as shown in Figure 4. Subsequent tests were carried out both for the sample before the absorption test, i.e., containing 70 wt.% electrolyte (sample HPE_4(70)), and after the absorption test, i.e., containing 87 wt.% electrolyte (sample HPE_4(87)).

### 3.4. Puncture Resistance

**Figure 5:**
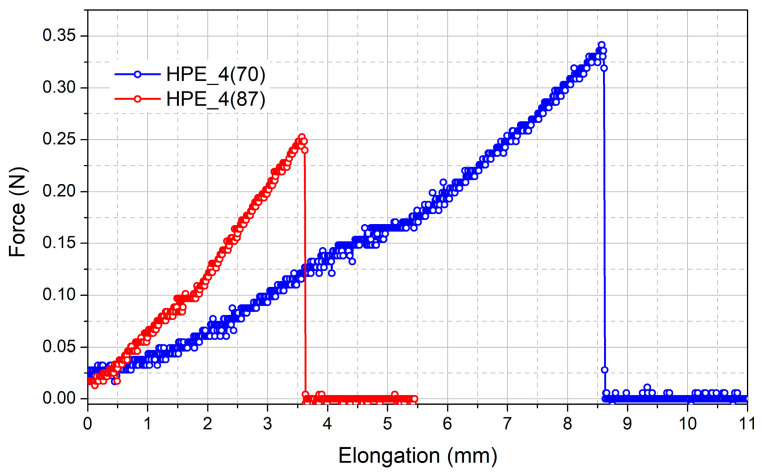
Force–elongation curves obtained from puncture resistance tests performed at room temperature (sample diameter: 13 mm, spherical probe diameter: 5 mm, test speed: 0.3 mm·s^−1^).

The puncture strength test was performed according to the previously described procedure, both for HPE samples immediately after polymerization and after they had been left for 24 h in a 1M ChNO_3_ solution. Example force-displacement curves are shown in Figure 5. During this test, a force is applied to the sample, and when a certain value is exceeded, the HPE is punctured by the probe, which is visible on the graph as a sudden break in the curve.

The value of the average force required to puncture the sample and its elongation along with the standard deviation are shown in Table 2.

The observed reduction in maximum force and elongation after electrolyte absorption results from the increased electrolyte content in the gel (from 70 wt.% to 87 wt.%). As is known from the literature [30], the mechanical properties of hydrogels are strongly influenced by the solvent volume fraction. This increase in solvent content leads to a decrease in mechanical strength and reduces its elasticity, which is associated with the penetration of solvent molecules into the polymer network. This penetration causes swelling of the polymer chains, increasing the distance between them and weakening intermolecular interactions, thereby reducing the integrity of the polymer matrix. Moreover, there is a smaller mass fraction of the polymer matrix per unit volume of the gel, which weakens its strength. Consequently, the stiffness of the hydrogel decreases due to the lower network density, making the material more susceptible to deformation and failure under applied stress, such as during puncture testing. Consequently, the mass fraction of polymer per unit volume decreases, weakening the hydrogel and increasing the mesh size of the polymer network in which the electrolyte is immobilized. Increased solvent content similarly causes swelling, greater distances between polymer chains, and weaker intermolecular interactions, making the material more susceptible to deformation and failure under applied stress. Nevertheless, it should be emphasized that HPE_4, both prior to and after electrolyte absorption, retained mechanical properties sufficient for practical handling, such as during conductivity measurements or capacitor assembly.

### 3.5. Thermal Characteristics

**Figure 6:**
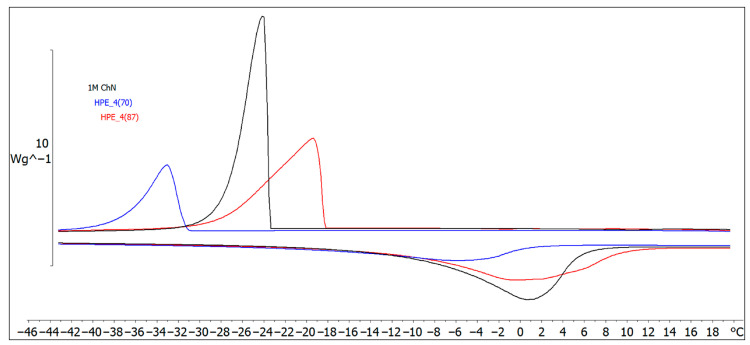
DSC thermograms of electrolyte (1M ChN) and hydrogels containing 70 wt.% electrolyte (HPE_4(70)) and 87 wt.% electrolyte (HPE_4(87)) (DSC, sample mass 5–10 mg, heating/cooling rate 10 °C·min^−1^, gas flow 20mL·min^−1^).

The electrolyte exhibits a crystallization temperature of −24.0 °C (Figure 6). In the hydrogel containing 70 wt.% of ChN solution, this temperature is shifted by approximately 10 °C toward lower values, reaching −32.5 °C. This effect can be attributed to the entrapment of the electrolyte within the hydrogel network and the disruption of molecular ordering caused by the polymer matrix, which delays the crystallization process. Moreover, the high flexibility of the hydrogel and the resulting mobility of the polymer chains may further contribute to the disturbance of structural ordering, thereby enhancing the system’s resistance to crystallization at low temperatures. An interesting result was obtained for the hydrogel containing 87 wt.% of the electrolyte, as in this case, the system crystallized at a higher temperature (−19.3 °C). This behavior suggests that the increased electrolyte content in the hydrogel reduces the degree of confinement within the polymer network, allowing for greater molecular mobility and partial restoration of the ordering necessary for crystallization. Consequently, the electrolyte behaves more like its bulk form, leading to a shift in the crystallization temperature toward higher values. The melting point, determined as the maximum of the transformation peak, of the tested samples ranges from approximately −6 °C to 1 °C. The lowest melting point of −6.0 °C was recorded for the HPE_4(70) hydrogel. The melting points of the pure electrolyte and the HPE__4(87) hydrogel are very similar and are 0.5 °C and −0.6 °C, respectively. In the case of the hydrogel containing 87% electrolyte, an additional melting transitions are visible as shoulders on the main transition peak at a higher temperature, around 2 °C and 6 °C, which indicate the presence of domains differing in the order of the structure or its reorganization.

### 3.6. Ionic Conductivity

The ionic conductivity of the electrolyte used in the study (1 M ChNO_3_ solution) was 60.5 mS·cm^−1^ and is comparable to aqueous solutions of other choline salts, e.g., the ionic conductivity of 1M ChBr is 50 mS·cm^−1^ [31], and 1 M ChCl is 60 mS·cm^−1^ [32]. Incorporating 70 wt.% electrolyte during gel synthesis led to a marked reduction in conductivity to 18.1 mS·cm^−1^ (Table 2), likely due to strong interactions between the polymer matrix and the electrolyte that hinder ion mobility. After electrolyte absorption, when electrolyte content increased to 87 wt.%—which, as noted above, led to a deterioration of mechanical properties—the ionic conductivity significantly increased to 34.2 mS·cm^−1^, thus exhibiting the opposite trend. This almost threefold improvement in ionic conductivity can be attributed to several factors. First, a higher solvent content per unit volume results in a greater concentration of mobile ions. Second, weakened interactions between HPE components at higher electrolyte content allowed for faster ion diffusion within the hydrogel, thereby increasing ionic conductivity. Therefore, factors that contribute to the deterioration of the mechanical properties of the polymer gel also contribute to an increase in its ionic conductivity.

**Figure 7:**
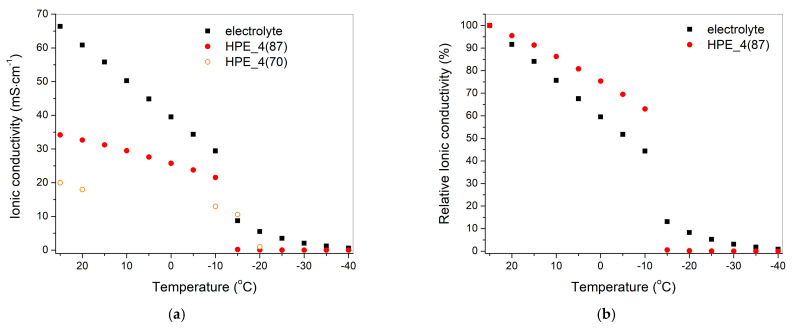
Dependence of (**a**) ionic conductivity and (**b**) relative ionic conductivity of the HPE_4(87) and pure electrolyte (1M ChNO_3_) on temperature (EIS, Swagelok^®^ two-electrode electrochemical cell, temperature range: 25 °C to −40 °C).

Given the potential use of the synthesized gels as separators in electrochemical capacitors, it is essential to maintain device operation at temperatures below 0 °C. The addition of choline salt is expected to lower the freezing point of water, enabling reliable operation of the energy storage device at low temperatures. Therefore, ionic conductivity measurements were performed over a temperature range of 25 °C to −40 °C for HPE_4(87) (thus after sorption of electrolyte) and for pure electrolyte. The results are presented in Figure 7. Additionally, the measurement results are presented in the form of relative ionic conductivity related to the highest measured value (at 25 °C) for HPE_4(87) and for pure electrolyte, in order to more clearly show the changes in conductivity depending on the temperature drop.

A sharp decrease in ionic conductivity was observed in tested samples as the temperature dropped below −10 °C. HPE_4(87) exhibited a particularly pronounced reduction in conductivity below this temperature (σ = 0.2 mS·cm^−1^). As shown earlier, this hydrogel crystallizes at −19.3 °C (DSC measurement); however, the conductivity measurements indicate that the onset of crystallization—leading to restricted ion mobility—occurs at a higher temperature. This behavior may be attributed to the presence of crystallization nuclei within the Swagelok^®^ cell, which could promote earlier crystallization of the sample. It is worth noting, however, that the conductivity did not drop to zero value, indicating that some degree of ionic diffusion persists within the material even at low temperatures. In contrast, the pure electrolyte sample showed a less abrupt decrease in conductivity (σ = 8.7 mS·cm^−1^), supporting the earlier assumption that crystallization in the electrolyte is incomplete, with a fraction of the electrolyte likely remaining in a liquid state. This observation prompted additional measurements for a hydrogel containing a lower electrolyte concentration, conducted over a narrower temperature range corresponding to the significant decline in conductivity. As shown, the conductivity of HPE_4(70) also decreased significantly, but the transition occurred at lower temperatures (around −20 °C). As previously discussed, this hydrogel exhibits a lower crystallization temperature compared to the other two samples. And the conductivity measurements confirm this result. Overall, these findings indicate that the tested electrolyte does not crystallize completely throughout the entire sample volume, but only partially. Consequently, the system maintains measurable ionic conductivity even below 0 °C, confirming the presence of residual ionic mobility within the hydrogel structure.

Moreover, it can be seen from Figure 7b that the HPE_4(87) system is characterized by a smaller decrease in ionic conductivity than the pure electrolyte. At −10 °C, the conductivity of HPE_4(87) is equal to 24.9 mS·cm^−1^, or 66% of the initial value, while that of the pure electrolyte is equal to 29.5 mS·cm^−1^, or 44% of the initial value. Thus, the decrease in conductivity for the hydrogel electrolyte is slower than for the liquid electrolyte. Comparing HPE_4(87) with the liquid electrolyte, the conductivity of the latter is 28.7 mS·cm^−1^ higher at 25 °C, whereas at –10 °C this difference decreases to only 4.6 mS·cm^−1^. This behavior likely arises from interactions between the polymer matrix and the electrolyte in the HPE: stronger interactions correspond to a smaller reduction in conductivity.

### 3.7. Electrochemical Investigation

**Figure 8:**
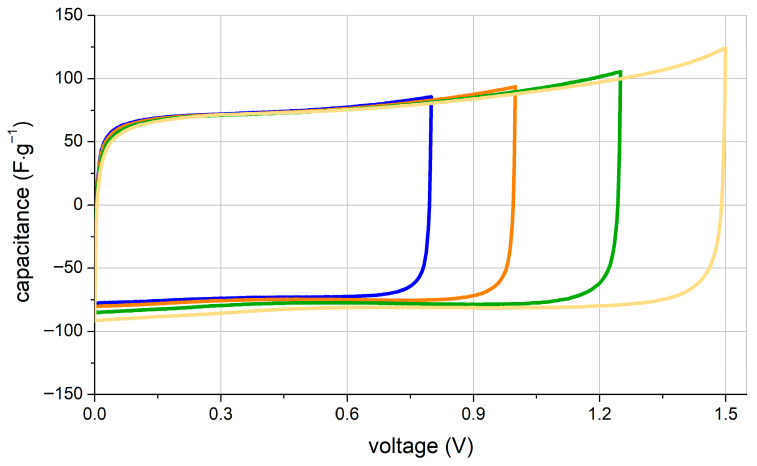
Cyclic voltammograms (CVs) of the AC/AC electrochemical capacitor recorded at various operating voltages (0.8–1.5 V), showing a nearly rectangular shape characteristic of EDLC behavior. Colors in the figure correspond to operating voltages: blue—0.8 V, orange—1.0 V, green—1.25 V, yellow—1.5 V.

**Figure 9:**
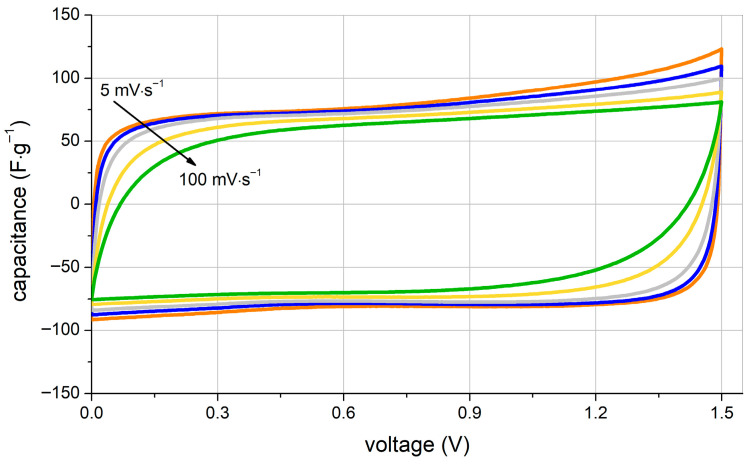
CV curves of the AC/AC electrochemical capacitor measured at different scan rates, demonstrating only slight deviation from the ideal rectangular profile even at 100 mV∙s^−1^. The colors in the figure correspond to different scan rates: orange—5 mV∙s^−1^, blue—10 mV∙s^−1^, gray—20 mV∙s^−1^, yellow—50 mV∙s^−1^, green—100 mV∙s^−1^.

The cyclic voltammograms (CVs) of the AC/AC electrochemical capacitor (Figure 8), recorded within the voltage range of 0.8–1.5 V, exhibit a nearly rectangular shape characteristic of electric double-layer capacitors (EDLCs). The absence of redox peaks on the CV curves confirms the purely capacitive behavior of the system. As shown in Figure 9, even when the scan rate is increased to 100 mV/s, only a slight deviation from the ideal rectangular profile is observed.

**Figure 10:**
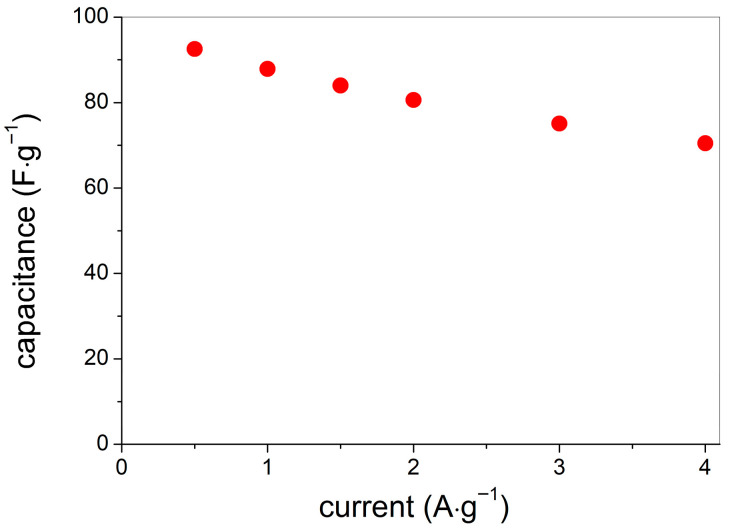
Specific discharge capacitance of the AC/AC electrochemical capacitor as a function of current density, indicating a gradual decrease in capacitance from ~93 F∙g^−1^ to ~71 F∙g^−1^ with increasing current.

The dependence of the discharge capacitance on the applied current density is presented in Figure 10. Increasing the current density from 0.5 A·g^−1^ to 4 A·g^−1^results in a decrease in specific capacitance from approximately 93 F·g^−1^ to around 71 F·g^−1^, corresponding to a reduction of about 34% relative to the initial value.

**Figure 11:**
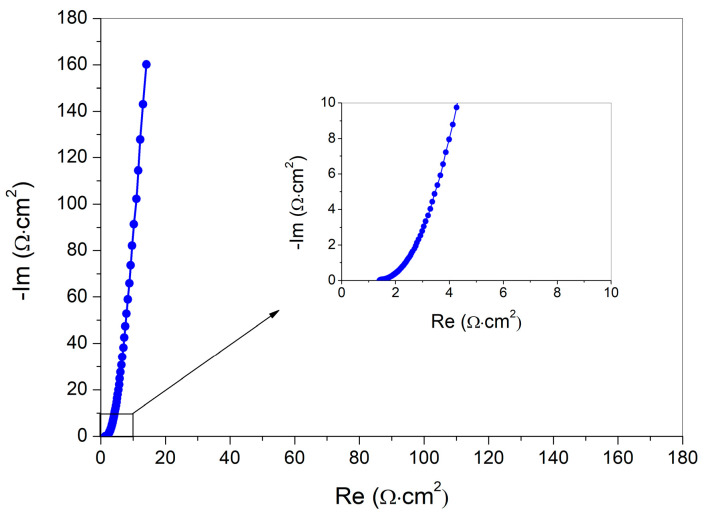
Nyquist plot of the AC/AC electrochemical capacitor with HPE electrolyte, showing an equivalent series resistance (ESR) of approximately 1.4 Ω∙cm^2^.

The Nyquist plot of the electrochemical capacitor (Figure 11) further reveals that the equivalent series resistance (ESR) of the device using the HPE electrolyte is approximately 1.4 Ω∙cm^2^.

**Figure 12:**
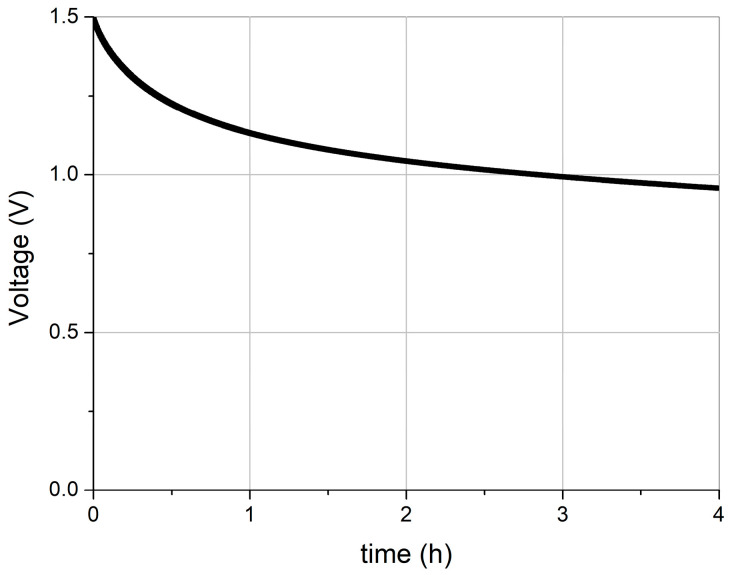
Self-discharge profile of AC/AC electrochemical capacitor with applied HPE.

The self-discharge test showed a noticeable voltage decay over the 4-h period (Figure 12). The capacitor, initially charged to 1.5 V, exhibited a gradual voltage drop to approximately 0.96 V, corresponding to a loss of about 36% of the initial voltage.

**Figure 13:**
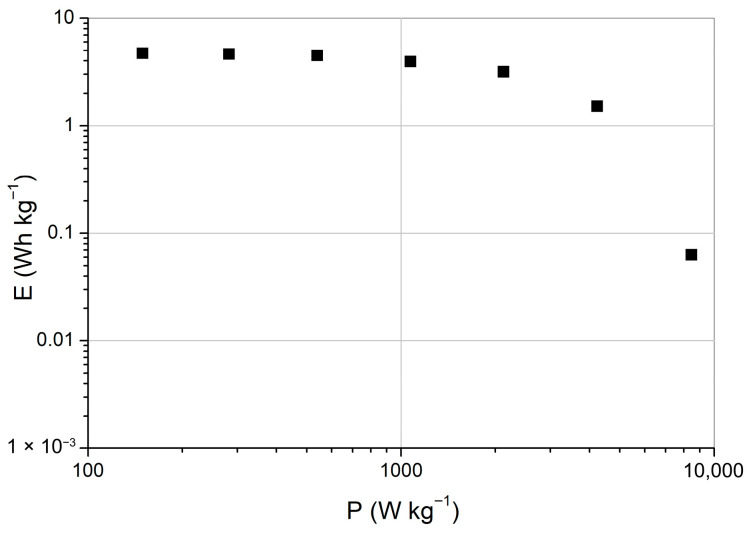
Ragone plot of the AC/AC capacitor with applied HPE.

The Ragone plot (Figure 13) demonstrates a typical trade-off between energy density and power density characteristic of EDLCs. At low power densities (around 150 W kg^−1^), the device exhibits an energy density of approximately 4.7 Wh kg^−1^. As the discharge rate increases, the available energy decreases gradually, reaching 3.2 Wh kg^−1^ at 2.1 kW kg^−1^ and dropping to 0.063 Wh kg^−1^ at the highest tested power of 8.5 kW kg^−1^. This behavior indicates that the capacitor maintains relatively stable energy output up to moderate power levels, but experiences pronounced losses at high discharge rates due to internal resistance and ion transport limitations. Overall, the results confirm that the device is well-suited for applications requiring rapid charge–discharge capability, although its energy storage is limited at high power outputs.

**Figure 14:**
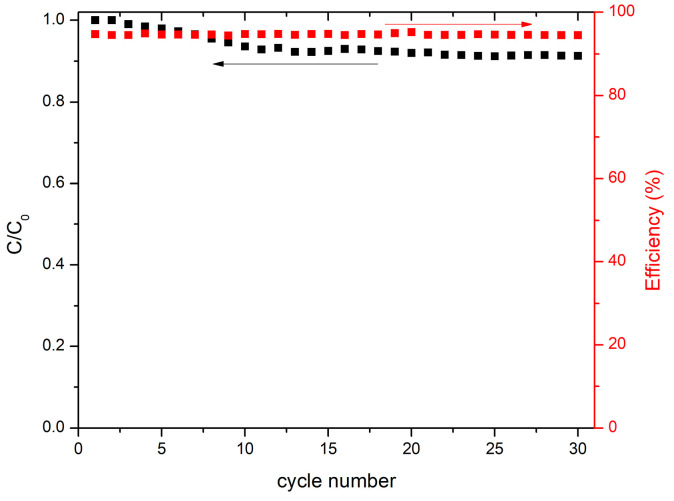
Retention of relative capacitance and charging/discharging efficiency during floating at 1.5 V of the AC/AC capacitor with applied HPE.

The floating test revealed a slow decrease in capacitance with cycling under constant voltage conditions. As can be seen from Figure 14 over 30 floating cycles, the relative capacitance (C/C_0_) decreased from 1.0 to approximately 0.91, corresponding to an overall loss of about 9% of the initial capacitance. The decline was most pronounced during the first ten cycles, after which the capacitance values tended to stabilize. This behavior suggests slow aging process, likely due to irreversible redox reactions, electrolyte decomposition, or structural rearrangement of the porous carbon matrix under prolonged polarization. Nevertheless, the retention above 90% after 30 cycles indicates satisfactory electrochemical stability of the device for short-term floating operation.

Overall, the obtained results demonstrate that the electrochemical capacitor with HPE exhibits excellent performance characteristics. The capacitance shows only a minor decrease with increasing scan rate and current density. Considering these findings, the application of HPE appears highly promising, particularly given the non-toxic nature of the electrolyte and its solid-state form, which eliminates the risk of leakage even in the event of mechanical damage to the capacitor.

## 4. Conclusions

By synthesizing a choline methacrylate monomer, it was possible to obtain, via in situ photopolymerization, a hydrogel polymer electrolyte exhibiting good compatibility between all components. The resulting hydrogel is transparent, with no evidence of phase separation or electrolyte leakage. The selected sample subjected to additional swelling in the electrolyte (HPE_4(87)) demonstrated sufficiently high ionic conductivity (~34 mS cm^−1^) to be considered suitable for energy storage applications. Moreover, the hydrogel exhibited favorable mechanical properties, allowing for manipulation during device assembly (e.g., in a capacitor) without structural damage. A capacitor fabricated with HPE_4 showed good operational performance. Accelerated aging tests showed that after the first 10 cycles of floating, the relative capacity dropped by approximately 8%, while after the next 20 cycles, this drop was only 1%, which indicates very good cyclic stability of the tested EDLC with HPE_4(87).

The HPE prepared in this work can be compared with other HPEs reported in the literature. Compared to hydrogels obtained by our group [33] using 1 M Na_2_SO_4_ electrolyte, the hydrogel developed in this work (1 M ChNO_3_) shows a comparable electrochemical performance (almost the same electrode material used) while offering several practical advantages. The 1 M Na_2_SO_4_-based gel achieves a specific capacitance of 120 F·g^−1^ at 0.5 A·g^−1^ and 70 F·g^−1^ at 4 A·g^−1^, but its ionic conductivity is much lower (15.5 mS·cm^−1^), and the synthesis process is more complex. In contrast, the 1 M ChNO_3_ system exhibits a conductivity of 34 mS·cm^−1^ and capacitances of 92.5 F·g^−1^ at 0.5 A·g^−1^ and 70.5 F·g^−1^ at 4 A·g^−1^, and is obtained by simple in situ polymerization of monomers with an electrolyte. These results indicate that despite the slightly lower capacitance at low current density, the ChNO_3_-based hydrogel offers better ionic transport and much simpler preparation, making it a more practical and efficient system. Other EDLCs with HPE presented in the literature [34,35] employ different electrode materials, and therefore a direct comparison of capacitance values is not entirely appropriate. Nevertheless, it is worth noting that the hydrogel presented by H. Wang et al. [34] shows the highest specific capacitance (157 F·g^−1^ at 1.5 A·g^−1^) and relatively high conductivity (48.8 mS·cm^−1^) but operates within a limited potential window of 0.8 V and requires a more complex synthesis. The HPE presented by Je et al. [35] achieves the highest ionic conductivity (68 mS·cm^−1^); however, its capacitance is markedly lower (24 F·g^−1^ at 1.0 A·g^−1^), and the synthesis procedure is much more demanding. In summary, the 1 M ChNO_3_-based hydrogel proposed in this work provides a well-balanced combination of ionic conductivity, electrochemical performance, and simplicity of synthesis, while ensuring good electrochemical parameters of EDLCs. Among systems using the same electrode material, it provides comparable capacitance and improved conductivity while maintaining an easy and scalable fabrication process suitable for quasi-solid-state supercapacitor applications.

## Figures and Tables

**Table 1:** Photocurable compositions used for the synthesis of hydrogel polymer electrolytes (HPE).

Sample	ChMAA, wt.%	PEGDA, wt.%	HEMA, wt.%	1M ChNO_3_, wt.%
HPE_1	10	5	15	70
HPE_2	10	3	17	70
HPE_3	10	1.5	18.5	70
HPE_4	10	1.2	18.8	70
HPE_5	10	1.1	18.9	70
HPE_6	10	1	19	70

**Table 2:** Force and elongation at puncture as well as ionic conductivity of the prepared hydrogel HPE_4 after polymerization (HPE_4(70)) and after sorption of electrolyte (HPE_4(87)).

	HPE_4(70)	HPE_4(87)
Force [N]	0.33 ± 0.02	0.28 ± 0.03
Elongation [mm]	7.9 ± 0.7	4.4 ± 1.1
Ionic conductivity [mS·cm^−1^]	18.1 ± 4.0	34.2 ± 1.0

## Data Availability

The data presented in this study are available upon request from the corresponding author.
